# Peripheral Nerve Ultrasonography in Chronic Inflammatory Demyelinating Polyradiculoneuropathy and Multifocal Motor Neuropathy: Correlations with Clinical and Neurophysiological Data

**DOI:** 10.1155/2016/9478593

**Published:** 2016-05-29

**Authors:** Aristide Merola, Michela Rosso, Alberto Romagnolo, Erdita Peci, Dario Cocito

**Affiliations:** Department of Neuroscience, University of Turin, Via Cherasco 15, 10126 Turin, Italy

## Abstract

*Objective.* This cross-sectional study analyzes the pattern of ultrasound peripheral nerve alterations in patients with chronic inflammatory demyelinating polyradiculoneuropathy (CIDP) and multifocal motor neuropathy (MMN) at different stages of functional disability.* Material and Methods.* 22 CIDP and 10 MMN patients and a group of 70 healthy controls were evaluated with an ultrasound scan of the median, ulnar, peroneal, tibial, and sural nerves. Results were correlated with clinical disability scales and nerve conduction studies.* Results.* Patients with intermediate functional impairment showed relatively larger cross-sectional areas than subjects with either a milder (*p* < 0.05) or more severe impairment (*p* < 0.05), both in CIDP and in MMN. In addition, MMN was associated with greater side-to-side intranerve variability (*p* < 0.05), while higher cross-sectional areas were observed in CIDP (*p* < 0.05) and in nerve segments with predominantly demyelinating features (*p* < 0.05). Higher CSA values were observed in nerves with demyelinating features versus axonal damage (*p* < 0.05 for CIDP; *p* < 0.05 for MMN).* Discussion and Conclusions.* Greater extent of quantitative and qualitative US alterations was observed in patients at intermediate versus higher functional disability and in nerves with demyelinating versus axonal damage. CIDP and MMN showed differential US aspects, with greater side-to-side intranerve variability in MMN and higher cross-sectional areas in CIDP.

## 1. Introduction

Multifocal motor neuropathy (MMN) and chronic inflammatory demyelinating polyradiculoneuropathy (CIDP) are acquired immune-mediated peripheral neuropathies (PN). MMN is a pure motor neuropathy syndrome usually beginning in one or both hands and principally affecting the upper extremities, characterized by a chronic or stepwise progressive asymmetrical limb weakness and muscle atrophy [1]. CIDP is an immune-mediated peripheral neuropathy that may cause weakness, paralysis, and/or impairment in both motor and sensory functions, usually affecting both sides of the body (symmetrical) [2].

The neurophysiological hallmark of MMN is conduction blocks (CB) outside the usual sites of nerve compression [1, 3], while CIDP typical features are CB, slowed motor, and sensory nerves conduction velocities and prolonged distal latencies [4]. In addition, both diseases may present a variable extent of axonal loss [5, 6], which has been attributed both to recurrent demyelinating insults and intrinsic pathogenic features, especially in the case of MMN [1].

Neuromuscular ultrasound (US) is a noninvasive, painless, and radiation-free complementary imaging technique for the diagnostic work-up of PN [7, 8]. Focal nerve enlargements can be observed in the majority of MMN patients and generalized nerve enlargements can be observed in CIDP patients, interestingly showing alterations also in limbs without signs of neurophysiological dysfunction [9–11]. However, the correlation between US, neurophysiological findings, and functional disability is still partially controversial [12–14]. Some authors have found an association between disease duration and the extent of nerve enlargement [15], while others have suggested a specific US pattern in relation to different mechanisms of injury [13]: demyelinating insults might result in swollen, enlarged, and hypoechoic nerves, while axonal damage may be characterized by hyperechoic atrophic bundles of fascicles. In addition, a variable combination of axonal and demyelinating insults may also coexist, resulting in hyperechoic and hypertrophic nerves. Most of literature data have been collected on CIDP patients, while a few sonographic-clinical-electrophysiological studies have been currently reported in MMN [9, 11, 16–18].

The aim of this study is to analyze US findings in patients with CIDP and MMN at different functional disability, in order to correlate US qualitative and quantitative measures with clinical and neurophysiological features.

## 2. Material and Methods

### 2.1. Subjects

This cross-sectional observational study includes 22 CIDP (5 females; 17 males) and 10 MMN (4 females; 6 males) patients recruited from the Neuromuscular Unit of Turin University Hospital between May 2014 and May 2015 and 70 healthy controls (43 men and 27 women).

Patients were evaluated by means of a clinical, US, electrophysiological assessment, and a structured clinical interview. All subjects fulfilled the European Federation of Neurological Society/Peripheral Nerve Society (EFNS/PNS) criteria for CIDP or MMN [3, 19] and, at the time of US examination, were receiving a monthly treatment with intravenous immunoglobulin (IVIg) (1-2 g/kg/month) for at least 6 months. Written informed consent and local ethical committee (*AOU San Giovanni Battista di Torino*) approval were obtained. The authors acted in accordance with the ethical standards laid down in the 1964 Declaration of Helsinki.

### 2.2. Clinical Assessment

A complete neurological examination was performed by means of the inflammatory neuropathy cause and treatment (INCAT) disability scale [20], the Medical Research Council (MRC) score in 8 muscle groups bilaterally (shoulder abduction, elbow flexion, wrist flexion, first dorsal interosseous, hip flexion, knee extension, and ankle flexion/extension), and the Overall Neuropathy Limitation Scale (ONLS).

According to the INCAT disability scale, upper limb activities were scored as “no/minimal impairment” (not affected), “moderate impairment” (affected but not prevented), or “severe impairment” (prevented), while the evaluation of walking difficulties was based on the use of aids: “no/minimal impairment” (no/minimal gait impairment); “moderate impairment” (moderate gait impairment, independent or with unilateral support); “severe impairment” (severe gait impairment, bilateral supports or wheelchair).

### 2.3. US Assessment

The US assessment was performed by means of a SonoSite M-Turbo Ultrasound Machine equipped with a broadband linear transducer (frequency band 6–15 MHz). The US scan was performed the same day of the neurological assessment by an evaluator (Michela Rosso), who was blinded to the clinical and neurophysiological data.

The following quantitative and qualitative US parameters were collected for the median, ulnar, peroneal, tibial, and sural nerves bilaterally.

*Nerve Cross-Sectional Area (CSA)*. CSA was measured by tracing the nerve just inside the hyperechoic rims with the “ellipse method” when applicable (when the nerve in the transverse scan had an elliptical or roundish shape) or alternatively tracing the nerve shape (when the nerve had an irregular shape) [13]. CSA was evaluated for each nerve at predetermined sites: median nerve was evaluated at wrist (entrance of carpal tunnel), middle third of the forearm, elbow (before penetrating the pronator teres muscle next to the brachial artery), and middle third of the arm (middle of the distance between medial epicondyle and axillary fossa); ulnar nerve at wrist (Guyon's canal), middle third of the forearm, elbow (between medial epicondyle and olecranon), and middle third of the arm (middle of the distance between medial epicondyle and axillary fossa); peroneal nerve at the fibular head and popliteal fossa; tibial nerve at popliteal fossa and at medial malleolus before its division into plantar nerves (ankle); sural nerve at the ankle. Maximal CSA (CSA^max^) enlargement was recorded for each nerve; median and ulnar nerves were scanned along the entire viewable tract, from the wrist to the middle third of the arm. Abnormal CSA values at entrapment sites (wrist, elbow, and fibular head) were excluded to avoid confounding local neuropathies.

*Intranerve CSA Variability*. Intranerve CSA variability was calculated as the ratio between CSA^max^/CSA^min^, for each nerve (available for median and ulnar nerves) [21].

*Side-to-Side Intranerve Variability*. Side-to-side intranerve variability was calculated as the “side-to-side” ratio of the intranerve CSA variability [12].

*Qualitative Analysis of Nerve Fascicles*. Nerves were classified as abnormal if ≥ 3 fascicles showed a cross-sectional area ≥ 2 mm^2^, regardless of the CSA value [22].

Normative US reference values were obtained by the assessment of healthy controls (Table 1), considering the upper threshold of the normality range (UT) to be the average value + 2 standard deviations. Then, in order to compare the CSA of different nerves taking into account their relative normative values, a normalized CSA (CSA^NORM^) was calculated by dividing the CSA^max^ of each nerve to the corresponding UT value (CSA^NORM^ = CSA^max^/CSA^UT^).

### 2.4. Nerve Conduction Studies

Nerve conduction studies were performed by means of a KeyPoint (Natus Medical Incorporated, San Carlos, CA, USA) electromyography (EMG) machine by evaluators blinded to the US study, assessing the bilateral peroneal, tibial, ulnar, and median motor nerves and the bilateral sural, median, and ulnar sensory nerves. Nerve conduction velocities (CV), compound muscle action potentials (CMAP), and sensory action potentials (SNAP) were collected and compared to the normality cut-off values of our laboratory; all patients were checked for skin temperature with a probe on the EMG machine. If needed, the body temperature was maintained above +34°C by means of an infrared lamp.

CB was defined in accordance with the EFNS/PNS criteria [3, 19], excluding possible sites of entrapment (wrist, elbow, and fibular head) to avoid confounding focal neuropathies. Moreover, neurophysiological alterations of nerve segments were stratified in predominantly “myelin damage” or “axonal damage” in accordance with the classification already proposed by Di Pasquale et al. [22].

### 2.5. Statistical Analysis

Descriptive statistics (mean, standard deviation, and range) were used for continuous variables. Mann-Whitney and Cramer's *V* tests were used for comparison between patients with different disease severity and neurophysiological alterations and between CIDP and MMN patients. Kruskal-Wallis test was applied for comparison among groups. Spearman's rho, Kendall's tau-b, and Pearson's correlations were used to estimate correlations between clinical, US, and electrophysiological characteristics, while a linear regression model was applied to estimate the influence of age, disease and treatment duration, and IVIg doses on CSA values. Bonferroni's correction for multiple comparisons was applied to avoid statistical biases of repeated testing effects. The average CSA values in bilaterally measured nerves were obtained pooling together data of the two sides. However, in order to take into account the asymmetrical involvement typical of MMN, we also considered the side-to-side intranerve variability, calculated by dividing the intranerve variability of the most affected side with the intranerve variability of the less affected side. All *p* values reported are two-tailed, considering 0.05 as statistical threshold. Analyses were performed with SPSS Statistics 21.0 for Mac.

## 3. Results

Complete clinical, US, and neurophysiological data were available for 22 CIDP and 10 MMN patients with similar age (62.7 ± 13.8 versus 55.1 ± 14.9 years old; *p*: 0.119) and disease duration (81.5 ± 60.0 versus 87.3 ± 46.6 months; *p*: 0.734). US data of 70 healthy controls (58.4 ± 16.1 years old; range 30–82) with normal clinical and neurophysiological assessments were used as normative reference values (Table 1).

### 3.1. Clinical and US Data

According to the INCAT disability score (Table 2), 7/32 subjects required a bilateral support/wheelchair (CIDP = 18%; MMN = 30%); 17/32 required a unilateral support (CIDP = 64%; MMN = 30%); and 8/32 did not show any significant impairment of gait (CIDP = 18%; MMN = 40%).

The upper limbs score showed that 9/32 subjects had a severe impairment in daily living activities (CIDP = 23%; MMN = 40%); 12/32 reported a moderate impairment (CIDP = 41%; MMN = 30%); and 11/32 did not report any significant impairment (CIDP = 36%; MMN = 30%). No differences were observed between CIDP and MMN patients at the INCAT (*p*: 0.519), ONLS (*p*: 0.724), and MRC (*p*: 0.327) scores (Table 2).

A total of 320 nerves (220 CIDP and 100 MMN) were evaluated by means of nerve conduction studies and US assessments: neurophysiological alterations were found in 78.0% of CIDP nerve segments (predominant myelin damage = 41.6%; predominant axonal damage = 36.4%) and in 62.5% of MMN nerve segments (predominant myelin damage = 35.0%; predominant axonal damage = 27.5%). Quantitative and/or qualitative US alterations were observed in 43.2% (95/220) of CIPD nerve segments and in 40.0% (40/100) of MMN nerve segments. In both cases these alterations were found more frequently in nerves with predominantly myelin versus axonal damage (CIDP = 74.6% versus 25% [*p*: 0.001]; MMN = 78.3% versus 20% [*p*: 0.010]). US abnormal features were additionally observed in 14.4% of CIDP and 10.1% of MMN nerve segments without significant neurophysiological alterations.

### 3.2. US Data in relation to Clinical/Neurophysiological Features

#### 3.2.1. Lower Limbs

**Figure 1:**
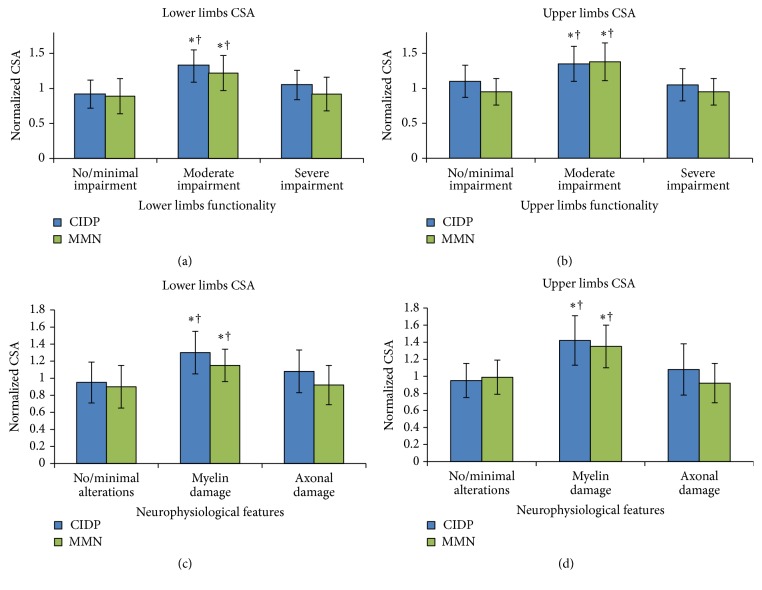
CSA values in relation to functional disability and neurophysiological alterations. Higher CSA values were observed in patients with intermediate functional disability and in nerves with predominant demyelinating features, both at the lower (a, c) and at the upper (b, d) limbs. Normalized cross-sectional area (CSA^NORM^) = maximal CSA of the nerve/upper threshold (UT) of the CSA normality range of the nerve (CSA^NORM^ = CSA^max^/CSA^UT^).  ^*∗*^Significant difference (*p* < 0.05) versus “no/minimal impairment” (a, b) or “no/minimal alterations” (c, d).  ^†^Significant difference (*p* < 0.05) versus “severe impairment” (a, b) or “axonal damage” (c, d). No/minimal impairment: INCAT = 0 (lower limbs) or 0-1 (upper limbs). Moderate impairment: INCAT = 1-2 (lower limbs) or 2 (upper limbs). Severe impairment: INCAT = 3–5 (lower and upper limbs).

As shown in Figure 1(a) and Table 3, CIDP and MMN patients with intermediate functional disability (gait disturbance that might require a unilateral support) showed higher CSA values than patients with no/minimal gait difficulties (*p*: 0.001 for CIDP and *p*: 0.002 for MMN) or higher functional disability (*p*: 0.041 for CIDP and *p*: 0.034 for MMN). Moreover, higher CSA values were observed (Figure 1(c)) in nerves with demyelinating features versus axonal damage (*p*: 0.048 for CIDP and *p*: 0.049 for MMN).

The quantitative US analyses showed higher CSA^max^ in CIDP than in MMN patients in peroneal nerve (16.81 ± 3.01 mm^2^ versus 13.60 ± 2.27 mm^2^; *p*: 0.024), tibial nerve (23.46 ± 2.23 mm^2^ versus 18.64 ± 2.66 mm^2^; *p*: 0.027), and sural nerve (3.56 ± 0.31 mm^2^ versus 2.60 ± 0.49 mm^2^; *p*: 0.047).

The qualitative US analyses revealed abnormal fascicles in 40% of MMN versus 22.7% of CIDP peroneal nerve segments (*p*: 0.171) and in 35% of MMN versus 15.9% of CIDP tibial nerve segments (*p*: 0.087).

Additionally, a significant correlation was found between abnormal nerve fascicles and CB (peroneal nerve: tau = 0.411 [*p*: 0.015] in CIDP and tau = 0.302 [*p*: 0.046] in MMN; tibial nerve: tau = 0.365 [*p*: 0.042] in CIDP and tau = 0.282 [*p*: 0.048] in MMN) and between abnormal nerve fascicles and CSA values, both in CIDP (peroneal nerve: rho = 0.329 [*p*: 0.033]; tibial nerve: rho = 0.296 [*p*: 0.049]) and in MMN (peroneal nerve: rho = 0.229 [*p*: 0.046]; tibial nerve: rho = 0.454 [*p*: 0.044]).

#### 3.2.2. Upper Limbs

**Figure 2:**
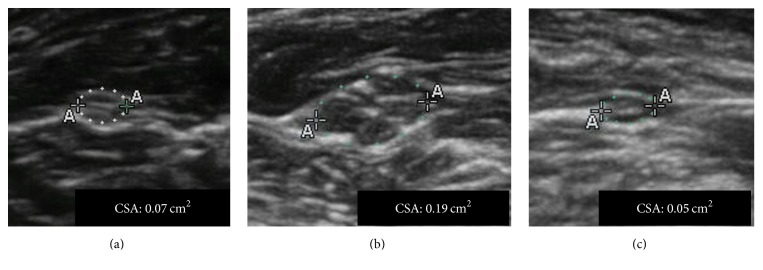
Median nerve axial ultrasound scan in CIDP patients at different disability state. (a) Male, 60 years old; disease duration 64 months; INCAT upper limbs score: 1. (b) Male, 63 years old; disease duration 72 months; INCAT upper limbs score: 2. (c) Male, 62 years old; disease duration 79 months; INCAT upper limbs score: 4.

Median and ulnar nerves CSA were significantly higher in patients with moderate impairment compared to subjects with either a more severe functional impairment (*p*: 0.037 for CIDP and *p*: 0.047 for MMN) or milder disability (*p*: 0.042 for CIDP and *p*: 0.037 for MMN) (Table 4, Figures 1(b) and 2).

The quantitative US analyses showed higher CSA^max^ in CIDP than in MMN patients in median nerve (18.70 ± 2.30 versus 14.85 ± 2.58; *p*: 0.042) and ulnar nerve (13.27 ± 2.64 versus 10.75 ± 2.23; *p*: 0.040), while the side-to-side intranerve variability was higher in MMN (median nerve: 1.9 ± 0.6 versus 1.5 ± 0.6 [*p*: 0.035]; ulnar nerve: 1.8 ± 0.4 versus 1.4 ± 0.4 [*p*: 0.007]) (Table 4).

The correlation between abnormal fascicles and CB was also confirmed for the upper limbs (median nerve: tau = 0.310 [*p*: 0.046] in CIDP and tau = 0.213 [*p*: 0.045] in MMN; ulnar nerve: tau = 0.260 [*p*: 0.049] in CIDP and tau = 0.271 [*p*: 0.048] in MMN), as was for the correlation between altered fascicles and CSA, both in CIDP (median nerve: rho = 0.315 [*p*: 0.037]; ulnar nerve: rho = 0.447 [*p*: 0.002]) and in MMN subjects (median nerve: rho = 0.331 [*p*: 0.043]; ulnar nerve: rho = 0.564 [*p*: 0.001]). Qualitative US analyses showed an inverse pattern compared to that observed at the lower limbs, with a moderately higher prevalence of altered fascicles in CIDP than in MMN nerve segments (median nerve: 38.6% versus 15% [*p*: 0.059]; ulnar nerve: 36.4% versus 20% [*p*: 0.194]). As observed in the lower limbs, the CSA values were higher in nerve segments with predominantly demyelinating features versus axonal damage (*p*: 0.001 for CIDP and *p*: 0.049 for MMN) (Figure 1(d)).

#### 3.2.3. Correlations between Clinical Features and Neurophysiological/US Data

There was a direct correlation between axonal damage and gait impairment at the lower limbs (CIDP: *r* = 0.456 [*p*: 0.002]; MMN: *r* = 0.450 [*p*: 0.036]) and between axonal damage and functional disability at the upper limbs (CIDP: *r* = 0.402 [*p*: 0.001]; MMN: *r* = 0.325 [*p*: 0.047]).

No linear correlations were observed between US data and INCAT score (CIDP: *r* = 0.121 [*p*: 0.110]; MMN: *r* = −0.239 [*p* = 0.190]) or between US data and ONLS score (CIDP: *r* = 0.053 [*p* = 0.415]; MMN: *r*: −0.211 [*p*: 0.140]), while an inverse correlation was observed in CIDP patients between MRC scores of muscles innervated by median and ulnar nerves and the corresponding intranerve variability (Table 5).

CSA values were not influenced by age (CIDP: *β* = 0.141 [*p*: 0.456]; MMN: *β* = 0.205 [*p*: 0.437]), disease duration (CIDP: *β* = −0.005 [*p*: 0.954]; MMN: *β* = −0.195 [*p*: 0.305]), treatment duration (CIDP: *β* = 0.027 [*p*: 0.749]; MMN: *β* = 0.295 [*p*: 0.247]), or IVIg dose (CIDP: *β* = 0.023 [*p*: 0.809]; MMN: *β* = 0.186 [*p*: 0.663]).

## 4. Discussion

This study reports the peripheral nerve US findings of 32 CIDP and MMN patients at different functional disabilities. Lower CSA values were associated with more severe clinical alterations and/or axonal damage, while higher CSA values were associated with intermediate functional disability (clinical alterations without loss of functionality) and/or demyelinating damage.

These data are in accordance with the findings reported by Di Pasquale et al. [22], who observed that nerve segments characterized by predominantly myelin damage had greater CSA than nerves with predominantly axonal damage and normal nerves, which virtually overlapped.

In addition, we found some differential aspects between MMN and CIDP: greater side-to-side intranerve variability was observed in MMN, in line with the pattern of heterogeneous and multifocal involvement characteristic of the disease; patients with CIDP showed higher CSA values, potentially indicating more prominent demyelinating processes; qualitative US analyses revealed a different distribution of abnormal fascicles in the upper and lower limbs, with more prominent US alterations in district affected by predominantly demyelinating damage (frequently associated with a less marked functional impairment) compared to district affected by secondary axonal degeneration.

The majority of literature data reported increased CSA values in CIDP, with a possible association between intranerve variability and functional scores [23, 24]. Less data are available for MMN, where asymmetric and focal CSA enlargements have also been reported in nerves without neurophysiological alterations, suggesting disease processes that could extend beyond the sensitivity of standard diagnostic techniques [9, 11, 16–18]. Several complex phenomena, such as segmental demyelination, proliferation of Schwann cells in response to repeated inflammatory insults, onion bulbs formation, and a variable degree of axonal loss might underlie these US findings [22, 25, 26]. However, their correlation with the mechanisms of nerve damage and repair still remains to be clarified.

Our data support the complementary role of US in the assessment of CIDP and MMN, suggesting a different pattern in nerves with demyelinating versus axonal damage and in CIDP versus MMN patients, in possible relationship with the different pathological processes involved.

Previous studies reported a correlation between disease duration and CSA values [14, 22, 27], while in our heterogeneous sample of patients we observed a “U-shaped” relationship between CSA values and functional impairment. We speculate that different disease phases might be associated with different US patterns, with an initial/intermediate phase of inflammation and myelin damage, characterized by increased CSA and enlarged swollen fascicles and a late phase of severe axonal degeneration, characterized by small atrophic fascicles, reduced CSA and greater functional impairment.

Other factors, such as IVIg pharmacological treatment and/or individual inflammatory response, might also be implicated in the morphological modifications of peripheral nerves [14]. However, the similar therapeutic regimen (IVIg) administered to our patients did not allow a post hoc analysis of treatment effects on CSA values. Finally, patients with CIDP, characterized by more prominent inflammatory and demyelinating features, might display greater nerve enlargement compared to MMN or to peripheral neuropathies characterized by primary axonal degeneration (i.e., chronic idiopathic axonal polyneuropathy).

Taken together, these findings suggest variable applications for US in the field of immune-mediated peripheral neuropathies, ranging from the more accurate clinicopathophysiologic phenotyping to the early detection of morphological changes associated with critical disease milestones. In addition innovative US score, such as the Bochum Ultrasound Score [10], will likely allow a more accurate differentiation between CIDP and other acquired or inherited peripheral neuropathies. However, US examinations require adequate training and experience to obtain reliable results.

## 5. Conclusions

Our findings suggest that CIDP and MMN patients with an intermediate functional disability may present more pronounced quantitative and qualitative US alterations than patients with higher disability. Moreover, some differential aspects can be recognized in CIDP versus MMN and greater US alterations might be observed in nerve segments with demyelinating versus axonal damage.

The strength of our findings is partially limited by the relatively small sample size and the lack of serial prospective follow-up assessments. In addition, two aspects should be considered in the interpretation of data: (a) the “U-shaped” relationship between US findings and functional impairment, which might result in a similar US pattern in patients with either minimal or severe disability; (b) the association of CSA reduction with two different factors (axonal damage and functional impairment), indicating the need of further prospective studies to analyze which of the two features primarily correlates with nerve size reduction.

## Figures and Tables

**Table 1:** Control group ultrasonographic data (70 subjects).

Nerve	Site	Average CSA (mm^2^)	Standard deviation
Peroneal	Popliteal fossa	8.91	1.82
	Fibular head	7.42	2.11

Tibial	Popliteal fossa	9.62	3.20
	Ankle	8.89	2.05

Sural	Ankle	2.15	0.62

Median	Wrist	8.07	1.30
	Middle third of the forearm	7.05	1.98
	Elbow	9.62	1.45
	Middle third of the arm	8.50	1.67
	CSA^max^	10.33	1.22
	Intranerve variability	1.76	0.47
	Side-to-side intranerve variability	1.27	0.17

Ulnar	Wrist	4.82	1.04
	Middle third of the forearm	6.07	1.42
	Elbow	5.94	1.82
	Middle third of the arm	7.31	1.79
	CSA^max^	8.75	2.09
	Intranerve variability	1.74	0.71
	Side-to-side intranerve variability	1.25	0.13

CSA: cross-sectional area.

**Table 2:** INCAT, ONLS, and MRC scores in CIDP and MMN patients.

		Upper limbs	Lower limbs	Total score
INCAT	CIDP	1.8 ± 1.0 (0–4)	1.3 ± 1.1 (0–4)	3.1 ± 2.0 (0–8)
	MMN	1.9 ± 1.0 (0–4)	1.2 ± 0.4 (1–3)	3.1 ± 0.9 (2–4)

ONLS	CIDP	1.9 ± 1.0 (0–4)	1.5 ± 1.4 (0–5)	3.4 ± 2.2 (0–9)
	MMN	1.9 ± 1.0 (0–4)	1.4 ± 0.7 (1–4)	3.3 ± 1.2 (2–5)

MRC	CIDP	36.9 ± 6.6 (15–40)	35.9 ± 6.6 (15–40)	72.8 ± 12.9 (30–80)
	MMN	36.0 ± 4.6 (25–40)	35.6 ± 4.4 (29–40)	71.6 ± 7.9 (60–80)

Results are reported as average ± standard deviation (minimum-maximum).

INCAT: inflammatory neuropathy cause and treatment.

MRC: medical research council.

ONLS: overall neuropathy limitation scale.

**Table 3:** Clinical, neurophysiological, and ultrasonographic data: lower limbs.

		No/minimal impairment (independent)	Moderate impairment (independent/unilateral support)	Severe impairment (bilateral support/wheelchair)	*p* value
*Peroneal nerve*					
Motor CV (m/sec)	CIDP	40.4 ± 1.3	26.7 ± 14.7^a^	21.0 ± 13.4	**0.006**
	MMN	40.7 ± 6.8	37.2 ± 5.5	33.9 ± 3.1	0.099

CMAP amplitude (mV)	CIDP	4.4 ± 3.0	1.7 ± 1.4^a^	0.1 ± 0.2	**0.016**
	MMN	3.1 ± 2.1	2.8 ± 1.8	1.1 ± 1.2	0.110

CSA popliteal fossa (mm^2^)	CIDP	12.2 ± 3.1	17.9 ± 3.5^a^	16.7 ± 2.0	**0.045**
	MMN	10.5 ± 1.5	17.3 ± 4.3	14.0 ± 1.9	0.114

CSA fibular head (mm^2^)	CIDP	8.8 ± 1.7	11.5 ± 3.0	11 ± 1.3	0.227
	MMN	7.8 ± 1.5	10.5 ± 2.3	9.3 ± 2.3	0.112

*Tibial nerve*					
Motor CV (m/sec)	CIDP	39.4 ± 6.6	32.7 ± 12.5	33.9 ± 0.9	0.121
	MMN	37.4 ± 3.6	34.6 ± 4.5	35.9 ± 4.0	0.234

CMAP amplitude (mV)	CIDP	3.9 ± 2.1	1.7 ± 2.4^b^	0.3 ± 0.1	**0.036**
	MMN	3.8 ± 1.1	2.3 ± 1.3	2.2 ± 2.7	**0.048**

CSA popliteal fossa (mm^2^)	CIDP	15.9 ± 3.8	26.7 ± 4.1	19.7 ± 3.9	**0.037**
	MMN	15.4 ± 3.9	23.8 ± 4.8	17.8 ± 2.2	0.241

CSA ankle (mm^2^)	CIDP	12.6 ± 2.1	15.7 ± 3.8	11.8 ± 2.5	0.254
	MMN	12.1 ± 2.4	14.5 ± 3.5^b^	8.7 ± 2.0	**0.032**

*Sural nerve*					
Sensory CV (m/sec)	CIDP	51.7 ± 3.3	25.1 ± 18.7	20.3 ± 18.3	0.255
	MMN	48.8 ± 7.8	45.4 ± 1.6	44.4 ± 4.3	0.441

SNAP amplitude (*μ*V)	CIDP	4.5 ± 2.3	3.4 ± 3.7	1.7 ± 2.9	0.111
	MMN	10.3 ± 6.1	9.9 ± 4.8	11.3 ± 5.4	0.638

CSA ankle (mm^2^)	CIDP	3.3 ± 0.3	3.7 ± 0.8	3.3 ± 0.2	0.928
	MMN	3.0 ± 1.1	2.7 ± 0.4	2.7 ± 0.5	0.958

Results are reported as average ± standard deviation.

CMAP: compound muscle action potential.

CSA: cross-sectional area.

CV: conduction velocity.

SNAP: sensory action potential.

INCAT (inflammatory neuropathy cause and treatment) lower limbs score: no/minimal impairment = 0; moderate impairment = 1-2; severe impairment = 3–5.

^a^Significant difference (*p* < 0.05) versus “no/minimal impairment.”

^b^Significant difference (*p* < 0.05) versus “severe impairment.”

**Table 4:** Clinical, neurophysiological, and ultrasonographic data: upper limbs.

		No/minimal impairment	Moderate impairment	Severe impairment	*p* value
*Median nerve*					
Motor CV (m/sec)	CIDP	39.6 ± 10.7	30.0 ± 14.9	29.5 ± 15.6	0.129
	MMN	48.9 ± 4.7	41.9 ± 13.9	40.5 ± 11.6	0.631

CMAP amplitude (mV)	CIDP	6.9 ± 3.3	3.2 ± 2.6^a^	2.2 ± 1.2	**0.007**
	MMN	6.7 ± 1.4	4.5 ± 3.3	3.3 ± 2.3	**0.034**

Sensory CV (m/sec)	CIDP	34.2 ± 23.3	31.1 ± 23.4	22.4 ± 22.2	0.509
	MMN	54.5 ± 4.4	55.8 ± 6.2	58.0 ± 8.1	0.738

SNAP amplitude (*μ*V)	CIDP	15.7 ± 23.7	4.2 ± 4.8	2.5 ± 3.7	0.236
	MMN	15.9 ± 8.1	18.0 ± 7.3	18.1 ± 9.3	0.267

CSA^max^ (mm^2^)	CIDP	17.8 ± 2.9	20.4 ± 5.7	14.1 ± 2.2	0.163
	MMN	12.2 ± 1.5	20.3 ± 4.5	12.8 ± 2.3	0.129

Intranerve variability	CIDP	2.9 ± 2.2	2.8 ± 1.7	2.0 ± 0.7	0.462
	MMN	2.3 ± 0.6	2.7 ± 1.5	2.1 ± 1.2	0.263

*Ulnar nerve*					
Motor CV (m/sec)	CIDP	43.0 ± 12.6	34.3 ± 9.5	29.8 ± 8.7	0.091
	MMN	50.7 ± 5.0	39.7 ± 6.5	36.7 ± 7.7	0.099

CMAP amplitude (mV)	CIDP	7.8 ± 2.9	4.6 ± 2.4^a^	3.8 ± 2.4	**0.004**
	MMN	5.6 ± 1.9	4.8 ± 2.2^b^	4.1 ± 3.8	**0.045**

Sensory CV (m/sec)	CIDP	44.6 ± 17.6	30.8 ± 18.8	26.8 ± 25.6	0.133
	MMN	47.6 ± 8.9	51.3 ± 7.6	51.6 ± 8.4	0.688

SNAP amplitude (*μ*V)	CIDP	17.7 ± 10.9	5.5 ± 4.2	2.2 ± 2.5	**0.031**
	MMN	15.5 ± 3.6	14.2 ± 8.9	9.7 ± 9.9	0.256

CSA^max^ (mm^2^)	CIDP	10.9 ± 2.9	14.4 ± 4.3^a^	11.9 ± 2.0	**0.038**
	MMN	10.0 ± 2.9	15.2 ± 4.8	9.5 ± 1.5	0.362

Intranerve variability	CIDP	2.4 ± 0.7	3.6 ± 1.2^a^	2.9 ± 0.7	**0.014**
	MMN	2.1 ± 0.7	3.7 ± 1.7^a^	2.2 ± 0.6	**0.043**

Results are reported as average ± standard deviation.

CMAP: compound muscle action potential.

CSA: cross-sectional area.

CV: conduction velocity.

SNAP: sensory action potential.

INCAT (inflammatory neuropathy cause and treatment) upper limbs score: no/minimal impairment = 0-1; moderate impairment = 2; severe impairment = 3–5.

^a^Significant difference (*p* < 0.05) versus “no/minimal impairment.”

^b^Significant difference (*p* < 0.05) versus “severe impairment.”

**Table 5:** Correlations between MRC score and corresponding ultrasonographic data in different muscles/nerves.

Muscle	Ultrasonographic data		Correlation coefficient	*p* value
Flexor carpi radialis (median nerve)	CSA^max^	CIDP	−0.197	0.223
		MMN	−0.107	0.654
	Abnormal fascicles	CIDP	−0.069	0.674
		MMN	−0.209	0.378
	Intranerve variability	CIDP	**−0.588**	**0.001**
		MMN	−0.002	0.993

First dorsal interosseous (ulnar nerve)	CSA^max^	CIDP	−0.176	0.276
		MMN	−0.020	0.932
	Abnormal fascicles	CIDP	−0.059	0.717
		MMN	−0.057	0.813
	Intranerve variability	CIDP	**−0.314**	**0.048**
		MMN	−0.050	0.834

Tibialis anterior (peroneal nerve)	CSA popliteal fossa	CIDP	−0.241	0.145
		MMN	−0.080	0.737
	CSA fibular head	CIDP	−0.371	0.228
		MMN	−0.014	0.954
	Abnormal fascicles	CIDP	−0.360	0.324
		MMN	−0.247	0.194

Gastrocnemius/soleus (tibial nerve)	CSA popliteal fossa	CIDP	−0.298	0.132
		MMN	−0.040	0.868
	CSA ankle	CIDP	−0.200	0.250
		MMN	−0.169	0.213
	Abnormal fascicles	CIDP	−0.267	0.146
		MMN	−0.190	0.423

CSA: cross-sectional area (mm^2^).

MRC: medical research council.
